# First transcriptome analysis of the venom glands of the scorpion *Hottentotta zagrosensis* (Scorpions: Buthidae) with focus on venom lipolysis activating peptides

**DOI:** 10.3389/fphar.2024.1464648

**Published:** 2024-11-13

**Authors:** Fatemeh Salabi, Hedieh Jafari, Masoud Mahdavinia, Reza Azadnasab, Saeedeh Shariati, Mahsa Lari Baghal, Majid Tebianian, Masoumeh Baradaran

**Affiliations:** ^1^ Razi Vaccine and Serum Research Institute, Agricultural Research, Education and Extension Organization (AREEO), Ahvaz, Iran; ^2^ Toxicology Research Center, Medical Basic Sciences Research Institute, Ahvaz Jundishapur University of Medical Sciences, Ahvaz, Iran; ^3^ Student Research Committee, Ahvaz Jundishapur University of Medical Sciences, Ahvaz, Iran; ^4^ Razi Vaccine and Serum Research Institute, Agricultural Research, Education and Extension Organization (AREEO), Karaj, Iran

**Keywords:** lipolysis activating peptide, *Hottentotta zagrosensis*, transcriptome analysis, venom gland components, pharmacological properties

## Abstract

**Introduction:**

Scorpion venom is a rich source of biological active peptides and proteins. Transcriptome analysis of the venom gland provides detailed insights about peptide and protein venom components. Following the transcriptome analysis of different species in our previous studies, our research team has focused on the *Hottentotta zagrosensis* as one of the endemic scorpions of Iran to obtain information about its venom proteins, in order to develop biological research focusing on medicinal applications of scorpion venom components and antivenom production. To gain insights into the protein composition of this scorpion venom, we performed transcriptomic analysis.

**Methods:**

Transcriptomic analysis of the venom gland of H. *zagrosensis,* prepared from the Khuzestan province, was performed through Illumina paired-end sequencing (RNA-Seq), Trinity *de novo* assembly, CD-Hit-EST clustering, and annotation of identified primary structures using bioinformatics approaches.

**Results:**

Transcriptome analysis showed the presence of 96.4% of complete arthropod BUSCOs, indicating a high-quality assembly. From total of 45,795,108 paired-end 150 bp trimmed reads, the clustering step resulted in the generation of 101,180 *de novo* assembled transcripts with N_50_ size of 1,149 bp. 96,071 Unigenes and 131,235 transcripts had a significant similarity (E-value 1e-3) with known proteins from UniProt, Swissprot, Animal toxin annotation project, and the Pfam database. The results were validated using InterProScan. These mainly correspond to ion channel inhibitors, metalloproteinases, neurotoxins, protease inhibitors, protease activators, Cysteine-rich secretory proteins, phospholipase A enzymes, antimicrobial peptides, growth factors, lipolysis-activating peptides, hyaluronidase, and, phospholipase D. Our venom gland transcriptomic approach identified several biologically active peptides including five LVP1-alpha and LVP1-beta isoforms, which we named HzLVP1_alpha1, HzLVP1_alpha2, HzLVP1_alpha3, HzLVP1_beta1, and HzLVP1_beta and have extremely characterized here.

**Discussion:**

Except for HzLVP1_beta1, all other identified LVP1s are predicted to be stable proteins (instability index <40). Moreover, all isoform of LVP1s alpha and beta subunits are thermostable, with the most stability for HzLVP1_alpha2 (aliphatic index = 71.38). HzLVP1_alpha2 has also the highest half-life. Three-dimensional structure of all identified proteins compacts with three disulfide bridges. The extra cysteine residue may allow the proteins to form a hetero- or homodimer. LVP1 subunits of *H. zagrosensis* potentially interact with adipose triglyceride lipase (ATGL) and hormone-sensitive lipase (HSL), two key enzymes in regulation of lipolysis in adipocytes, suggesting pharmacological properties of these identified proteins.

## 1 Introduction

Scorpion envenomation as a life-threatening emergency is one of the most important health challenges in tropical and sub-tropical countries of the world (Chippaux, 2015; Ebrahimi et al., 2017; Kazemi et al., 2024; Kumar, 2022; Lacerda et al., 2022; Mabunda et al., 2024). Every year in Iran, despite proper treatment, twenty deaths resulting from 42,500 cases of scorpion stings have been reported (Dehghani and Fathi, 2012; Baradaran et al., 2024). Previous studies indicated that Iranian scorpion fauna consists of 89 species belonging to 20 genera from four families; Buthidae, Scorpionidae and Hemiscorpiidae, Diplocentridae, with the most prevalent (88.7%) of Buthidae family (Amiri et al., 2024; Barahoei, 2022; Barahoei et al., 2022; Kazemi and Sabatier, 2019; Kovařík, 2019; Kovařík and Navidpour, 2020; Kovařík et al., 2020; Kovařík et al., 2022). *Hottentotta zagrosensis,* a scorpion species belonging to the Buthidae family, is an endemic species to Iran (Fet et al., 2000). This species is a non-digging scorpion, entirely black in color, that prefers mountainous and rocky area habitats. It occurs in the Zagros chain region in Fars, Khuzestan, Kohgilouyeh va Boyer- Ahmad, Lorestan, and West Azerbaijan provinces, and lives underground in tunnels excavated in the soil (Rafinejad et al., 2020).

Recently, understanding the diversity and composition of animal venoms has been pursued through analysis of the transcriptome or the proteome (Baradaran et al., 2011; Lüddecke et al., 2023; von Reumont et al., 2022). The data obtained from the analysis of the transcriptomes of the venom glands of scorpions, in addition to improving our knowledge about the diversity of the heterogeneous venom components, inspires more research on these compounds. In the case of deadly scorpions, it can contribute to the eventual production of specific antibodies by identifying the toxins with lethal effects (Kazemi-Lomedasht et al., 2017; Rivera-de-Torre et al., 2024; Santibáñez-López et al., 2016). Transcriptome annotation and characterization of scorpion species, including *Androctonus mauretanicus, Babycurus gigas, Grosphus grandidieri, Hottentotta gentili, Protoiurus kraepelini*, and *Nebo hierichonticus* have revealed novel toxins, including non-disulfide-bridged and disulfide-bridged toxins (Grashof et al., 2019). Analysis of the transcriptome from the venom gland of scorpion *Mesobuthus martensii* revealed diverse expression of scorpion toxin genes (Yang et al., 2024). Additionally, transcriptome analysis revealed the presence of important elements of the small non-coding RNA processing machinery, as well as microRNA candidates in the venom gland of *Centruroides noxius* Hoffmann (Rendón-Anaya et al., 2012). Using transcriptome analysis of venom glands, a total of 122,421 mRNAs have been identified from venom gland libraries of the scorpion *Androctonus crassicauda* (Salabi et al., 2021a). Several isoforms of enolase (Pondehnezhadan et al., 2023), hyaluronidase (Salabi and Jafari, 2023), PLD (Baradaran and Salabi, 2023) and PLA_2_ families (Salabi and Jafari, 2024) have been identified from venom gland libraries of the scorpion *Androctonus crassicauda*, *Hemiscorpius lepturus*, *Hottentotta saulcyi*, and *Mesobuthus eupeus*. The main advantage of venom transcriptome research is mainly focused on the study of venom composition through transcriptome data, which allows the study of expression patterns of venom-encoding genes (Salabi and Jafari, 2022). Following the previous research on transcriptome analysis of scorpions living in Khouzestan province, as a province with the most scorpion stings and scorpion variation in Iran (Baradaran et al., 2018; Baradaran and Salabi, 2023; NaderiSoorki et al., 2016; Salabi and Jafari, 2024; Salabi et al., 2023), we selected *H. zagrosensis*, an endemic species, for transcriptome analysis and *in silico* identification of putative venom genes expressed in the venom glands.

LVP1 subunits (alpha and beta) have been identified in the venom of different scorpion species, including *Buthus occitanus tunetanus* (Soudani et al., 2005b; Zhu and Gao, 2006), *Mesobuthus martensii* (Chai et al., 2012; Nie et al., 2012), *Lychas mucronatus* (Ruiming et al., 2010), *H. saulcyi*, *M. eupeus*, and, *A. crassicauda* (Baradaran and Salabi, 2023). Lipolytic properties of LVP1, which has been proven in previous studies to stimulate lipolysis in rat adipocytes, has caused this peptide to be considered as a candidate for a drug that regulates serum cholesterol (Chai et al., 2012; Drira-Chaabane et al., 1996; Soudani et al., 2005a). A reduction in total cholesterol in canines injected with scorpion venom confirms the presence of cholesterol-regulating components in the venom gland of scorpions (Murthy and Medh, 1986). LVP1 beta subunit can inhibit the activity of 3-hydroxy-3-methylglutaryl-coenzyme A (HMG-CoA) reductase, the regulator enzyme of cholesterol biosynthesis pathway, compared to simvastatin or atorvastatin, two main drugs for treatment of hypercholesterolemia (Chai et al., 2012).

To provide more useful information for scorpion venom studies, this study analysed the transcriptome of H. zagrosensis focusing on lipolysis activating peptides (LVP).

## 2 Materials and methods

### 2.1 Sample preparation and RNA extraction

The venom gland tissues were obtained from ten specimens of *H. zagrosensis*, 2023, collected from deserts of Baghmalek on southwest of Khuzestan providence, Iran. The venom glands were removed 3 days after venom harvesting by electrostimulation. All experiments were performed in accordance to the ethical principles and the national norms and standards for conducting Medical Research in Iran and were authorized by Ahvaz Jundishapur University of Medical Sciences (Ethical code: IR.AJUMS.REC.1400.556) and the Institutional Animal Care Committee of Razi Vaccine and Serum Research Institute (Permit number IR.RVSRI.REC.1401.017). Thereafter, the venom gland tissues of *H. zagrosensis* individuals were powdered in liquid nitrogen and RNA extraction was done using the RNeasy Animal Mini Kit (Qiagen, Valencia, CA, United States) according to the manufacturer’s instructions. The RNA quality was assessed for all samples by gel electrophoresis and the RNA concentration was measured by Nanodrop (co. Thermo, United States).

### 2.2 cDNA library construction

Six of the best samples of *H. zagrosensis* venom gland tissues were selected and two groups of three were pooled together in equal concentrations to generate two pooled RNA samples. The RNA Integrity Number (RIN) of samples were determined by Macrogen, Inc (Seoul, Korea) using Agilent 2100 Bioanalyzer System (Agilent Technologies, United States) according to manufacturer’s instructions. The RNA samples with a RIN >7 were selected for cDNA library construction. The cDNA library was sequenced using a high-throughput RNA-sequencing Illumina HiSeq 2000 platform (Macrogen Co. Macrogen, Seoul, Korea), with 150 bp paired-end reads. The quality measurement of the reads was done using FastQC software v0.11.5 (Wingett and Andrews, 2018) with default parameters. To remove low-quality reads and any Illumina adapter remnants, the raw reads were subjected to the Trimmomatic software v2.10.0 (Bolger et al., 2014) with settings detailed in our previous work contribution (Baradaran and Salabi, 2023). Finally, we reused the FastQC to verify the final quality of the dataset.

### 2.3 Assembly of read-sequences and bioinformatics analysis

After trimming and assessing the quality of raw reads, the trimmed reads of two pooled samples were assembled *de novo* by Trinity v2.15.1 (https://github.com/trinityrnaseq/trinityrnaseq/releases) using the following parameters: --normalize_reads, --seqType fa, --SS_lib_type RF, --max_memory 32G, --CPU 8. Then, clustering the resulted sequences was performed using the comprehensive clustering package of CD-HIT-EST v4.7 (Li and Godzik, 2006) to reduce sequence redundancy. The quality of the assembly generated from Trinity only (Raw assembly) and the assembly resulting from the clustering step (Final assembly) were analyzed with Trinity script “TrinityStats.pl” of the Trinity toolkit. The completeness of assembles were measured by counting the percentage of orthologues conserved across Arachnida and Arthropoda databases using BUSCO (Benchmarking Universal Single-Copy Orthologs) package v5.2.2 (Li and Godzik, 2006). To further assess the quality of the raw and clustered *de novo* assemblies, we mapped cleaned reads back to their corresponding assemblies using Bowtie2 v2.3.4.1 (Langmead and Salzberg, 2012). Finally, we used Croco v0.1 software (Bergmann et al., 2016) to assess the pervasive cross-species contamination in our assemblies. We subsequently used the TransDecoder v5.5.0 program with the “single best ORF” option to predict the gene Open Reading Frames (ORFs) or protein coding regions (https://github.com/TransDecoder/TransDecoder/releases) within the assembled transcriptome. TransDecoder used by default the universal genetic code. The Trinotate v4.0.2 was used to annotate the venom gland assembled transcriptome.

Full-length transcripts harboring protein domains in the *de novo* assembled transcriptome of the *H. zagrosensis* were identified using BLASTX or BLASTP by alignment against the Swiss-Prot, UniProtKB/TrEMBL, and Pfam protein domain databases with an *E*-*value* threshold of 10^–3^. Furthermore, the venom proteins and toxins in the *H. zagrosensis* venom gland transcriptome were identified using BLASTP with an *E*-*value* threshold of 10^–3^ against the customized databases of manually reviewed venom proteins and toxins obtained from Animal toxin annotation project (https://www.uniprot.org/program/Toxins) (Release 2023_05) of Uniprot KB database as a reference. We used the Venn diagram (http://bioinformatics.psb.ugent.be/webtools/Venn/) to visualize the blast results.

### 2.4 Extraction and classification of lipolysis activating peptides genes

We followed the procedure described in our previous studies to identify members of the lipolysis activating protein family (Salabi et al., 2023). In brief, using BLAST searches of the NCBI database, the cDNAs and amino acids sequences representing the Lipolysis activating peptide annotation were collected from scorpions and closely related species. A local database of lipolysis activating proteins was created, which were used as queries for BLAST searches of the *H. zagrosensis* transcriptome. This database was constructed by gathering known LVP1 sequences from various scorpion species as well as our previously work from scorpions of *Mesobuthus eupeus*, *Hottentotta saulcyi*, and *Androctonus crassicauda* (Baradaran and Salabi, 2023), and closely related species including spiders, ticks, mites, termites, ants, flies, and wasps. Several sequences representing the lipolysis activating proteins annotation were identified, then extracted from cDNA libraries of *H. zagrosensis* venom glands, and grouped based on their similarity to known LVP proteins. All sequences of LVP proteins found in this study have been deposited in the GeneBank database. The InterProScan web service (https://www.ebi.ac.uk/interpro/search/sequence/), which scans the InterPro database for matches, was then used to perform functional analysis of all data and also LVP1 proteins, separately based on predicting domains and essential sites encoded by the venom glands and classifying them into families.

Amino acids alignment of identified LVPs with homologous sequences were done using MAFFT program version 7 (https://mafft.cbrc.jp/alignment/server/index.html). The SignalP 6.0 server (https://services.healthtech.dtu.dk/service.php?SignalP) was used to predict the signal peptides and the location of their cleavage sites in LVP1 proteins from *H. zagrosensis* venom glands. Then the predicted mature protein sequences of LVP1 retrieved from *H. zagrosensis* venom gland transcriptome dataset were subjected to computation for various physico-chemical parameters using Expasy’s ProtParam server (available at https://web.expasy.org/protparam/) and INNOVAGEN (http://www.innovagen.com/proteomics-tools).

### 2.5 Gene ontology and pathway enrichment analysis

The LVP coding genes were analysed for Gene Ontology (GO) and the Kyoto Encyclopedia of Genes and Genomes (KEGG) pathways (http://www.kegg.jp/kegg/kegg1.html) to investigate the main functions of those mRNAs. The GhostKOALA server (http://www.kegg.jp/ghostkoala/) as a KEGG automatic annotation server and Reconstruct Pathway (http://www.kegg.jp/kegg/tool/map_pathway.html) as KEGG Mapper tool were applied for the alternative functional annotation of protein isoform sequences resulted from TransDecoder by their associated biological pathways.

### 2.6 RNA–RNA interaction prediction

We applied RNAplex v1.6 (Tafer and Hofacker, 2008) to measure the minimum free energy between secondary structure of two RNA molecules; mRNAs representing LVP1s and other sequences from assembly of *H. zagrosensis* venom gland transcriptome, using -e -25 parameter. Then, interacting RNAs with minimum free energy smaller than −25 kcal/mol were considered as stable interactions and they were kept for more analysis. Furthermore, in order to gain insights into the functional annotation and biological pathways where each interacted sequence could be involved, the selected sequences were submitted to KEGG automatic annotation web server (http://www.kegg.jp/ghostkoala/).

### 2.7 Structural homology analysis of LVP1 isoforms

For molecular modeling of VLP isoforms, the amino acid sequences of lipolysis activating proteins from *H. zagrosensis* were submitted to the SWISS-MODEL (http://swissmodel.expasy.org) and I-TASSER (https://zhanggroup.org/I-TASSER/) online software to predict and build the tertiary structural model of lipolysis activating peptides. The quality of the predicted structures was evaluated via ERRAT, Verify3D and PROCHECK through the SAVESv6.0 (https://saves.mbi.ucla.edu/) and also by Z-score through the ProSA-web (https://prosa.services.came.sbg.ac.at/prosa.php). The predicted three-dimensional structures were used for structural comparison between alpha and beta LVPs derived from *H. zagrosensis*. The UCSF Chimera software (ver. 1.11.2, University of California, San Francisco, CA, United States) was used for structural alignment and visualization of the predicted structures of alpha and beta LVP isoforms. UCSF Chimera software is a program for the interactive visualization and analysis of molecular structures (https://www.cgl.ucsf.edu/chimera/).

## 3 Results

### 3.1 Sequences assembly and completeness

Illumina paired-end sequencing of cDNA constructed from venom gland RNA samples of *H. zagrosensis* with RIN values higher than 7. After adapter and low-quality reads trimming, 45,795,108 paired-end 150 bp trimmed reads were obtained. Trinity *de novo* assembling produced 213,626 contigs greater than 200 bp in length, including variant isoforms per contig. The contigs were assembled into 136,897 Trinity genes with a median transcript length 481 (Table 1). To evaluate the quality of the resulted transcriptomes, the cleaned reads first were mapped back to their corresponding assembly using Bowtie2 v2.3.4.1, which resulted to > 98.93% mapped reads.

**TABLE 1:** Summary statistics of raw and clustered *de novo* transcriptome assemblies for the *H. zagrosensis* venom gland.

	Raw assembly	Final assembly
Total trinity transcripts	213,626	101,180
Total trinity “genes”	136,897	96,071
Annotated unigene in databases	20,345	20,345
GC%	32.87	32.02
Total number of assembled bases	224,089,244	68,000,103

Raw Assembly: The *H. zagrosensis* assembly generated from Trinity only.

Final Assembly: The *H. zagrosensis* assembly, resulting from the clustering step.

The statically analysis of clustered transcriptome assembly showed that the *de novo* assembly of all reads resulted in a total assembly of 68,000,103 bp representing 101,180 transcripts with N_50_ size of 1,149 bp and corresponding to 96,071 Trinity genes. Cd-Hit-EST clustering reorganized the Trinity genes into 101,180 contigs, i.e., clustering transcripts into putative genes reduced the putative gene number by 29.82% compared to raw assembly using Trinity only (Tables 1, 2).

**TABLE 2:** Assessing the quality of the raw and clustered *de novo* transcriptome assemblies of the *H. zagrosensis* venom gland.

	Situation of contigs according to			
	All transcript contigs		Longest isoform per unigene	
	Raw assembly	Final assembly	Raw assembly	Final assembly
Contig N10	5,229	4,403	4,008	4,142
Contig N20	4,072	3,127	2,836	2,865
Contig N30	3,299	2,279	2,077	2,073
Contig N40	2,660	1,670	1,515	1,475
Contig N50	2,116	1,149	1,044	969
Median contig length	481	345	342	335
Average contig	1,048.98	672.07	645.95	625.89

Data are provided for all transcripts or for the longest isoform per unigene.

Detailed results of calculation of the number of complete, duplicated, fragmented, and missing transcripts in each of our *de novo* assemblies of *H. zagrosensis* venom gland against the Arthropoda and Arachnida gene databases are shown in Table 3. Of the 1,013 conserved Arthropod genes and 2,934 conserved Arachnida genes in the BUSCO database, our raw assembly contained 977 and 2,722 complete genes, respectively. The BUSCO analysis showed a high BUSCO completeness score of >92%, and a high level of duplicated matches to the BUSCO sequences for the *de novo* raw assembly of *H. zagrosensis* venom gland transcriptome, consisting of 90.9% and 85.1% complete duplicated BUSCOs and 5.5% and 7.7% complete single-copy orthologues of the Arthropoda and Arachnida data set, respectively. To achieve a less redundant high-quality transcriptome, we used the CD-HIT-EST program. We further compared completeness of raw assembly to final assembly. Interestingly, the clustering step decreased the level of duplicated sequences against both Arthropoda and Arachnida databases. The resulting final transcriptome contained 19.2% and 20.7% complete duplicated copies, and 76.5% and 71.0% complete single-copy orthologues of the Arthropoda and Arachnida BUSCO data sets, respectively (Table 3). We used Croco v0.1 software (Panda and Chandra, 2012) to verify that the resulting redundancy was not due to pervasive cross-species contamination in next-generation sequencing data. The results showed no contamination.

**TABLE 3:** BUSCO analysis using default parameters for raw assembly and final assembly of *H. zagrosensis* venom gland with the Arthropoda_odb10 and Arachnida_odb10.

Samples name	Cross Arthropoda_odb10		Cross Arachnida_odb10	
	Raw assembly	Final assembly	Raw assembly	Final assembly
Complete BUSCOs	96.4%	95.7%	92.8%	91.7%
Single-copy BUSCOs	5.5%	76.5%	7.7%	71.0%
Duplicated BUSCOs	90.9%	19.2%	85.1%	20.7%
Fragmented BUSCOs	1.9%	2.4%	2.4%	3.0%
Missing BUSCOs	1.7%	1.9%	4.8%	5.3%
Number of genes	1,013	1,013	2,934	2,934
Total BUSCOs groups searched	1,013	1,013	2,934	2,934

Raw Assembly: The *H. zagrosensis* assembly generated from Trinity only.

Final Assembly: The *H. zagrosensis* assembly, resulting from the clustering step.

Single: Complete BUSCOs represented by one transcript.

Duplicated: Complete BUSCOs represented by more than one transcript.

Fragmented: Partially recovered BUSCOs transcripts.

Missing: Not recovered transcripts.

Moreover, the analysis of Arthropoda BUSCO scores for the final assembly was done which the results are presented in Table 3.

### 3.2 Annotation

A total of 62,040 protein coding isoform sequences was predicted, of which signal peptides were found for 5,386 peptides and proteins. Functional annotation revealed that among the predicted protein sequences, 64% were annotated, and the most of these genes encode proteins involved in the genetic information processing of the cell. It also confirms that these sequences are related to scorpions because most of the sequences are similar to arthropods (Supplementary Figure S1).

**FIGURE 1:**
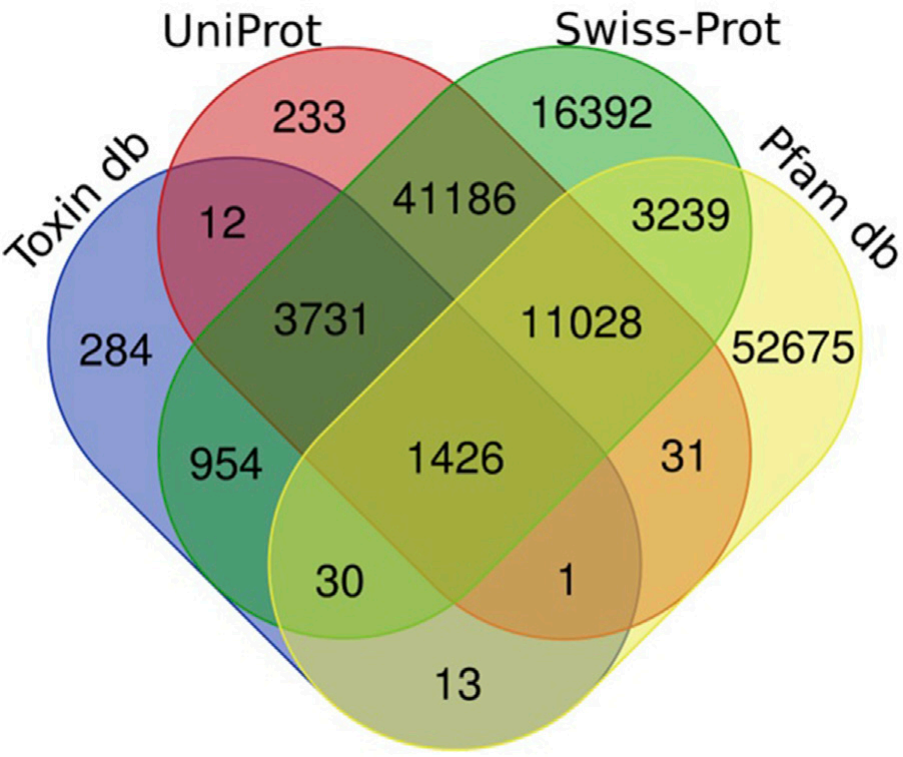
Number of unigenes of *H. zagrosensis* venom gland components matched to UniProt, Swissprot, Animal toxin annotation project (Toxin db), and Pfam databases. The overlap regions show number of matched common unigenes between databases.

Sequence homology searches using the Basic Local Alignment Search Tool (BLAST) revealed that 131,235 transcripts had a significant similarity (e-value 1e-3) with known proteins from UniProt, Swissprot, Animal toxin annotation project, and Pfam databases (Figure 1). The Venn diagram of Figure 1 shows that the largest number of transcripts (77,986) were matched to Swissprot. Of these, 61,594 sequences overlapped with other databases and 16,392 sequences had only significant BLAST alignments to the SwissProt database. The similarity searches showed that 68,443 transcripts were matched to Pfam, and of these, 15,732 sequences overlapped with other databases and 52,720 sequences had only significant BLAST alignments to the Pfam database. Annotation against the UniProt database showed that out of 57,648 transcripts that had significant similarity to known proteins in this database, and 57,415 sequences overlapped with other databases; 233 sequences show only similarity to UniProt.

**FIGURE 2:**
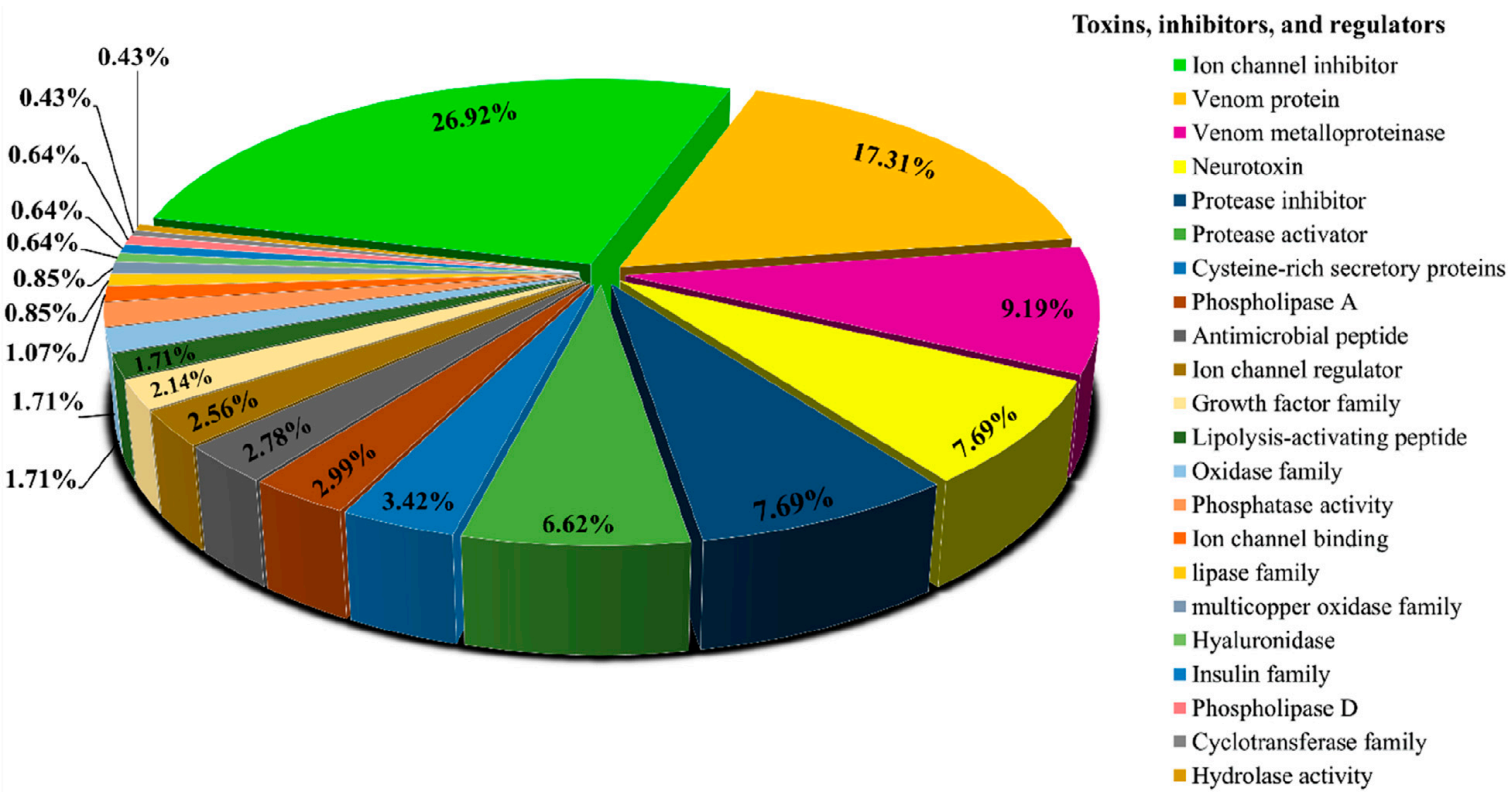
Functional classification of protein toxins and protein families identified in *H. zagrosensis* venom using Animal toxin annotation project as reference.

Searching the predicted proteins against all of the sequences from the Animal Toxin Annotation Project (https://www.uniprot.org/program/Toxins) revealed that, out of 6,451 transcripts that had significant similarity to the Animal toxin annotation project database, 284 sequences only found to have similarity with this database. Figure 2 shows the classification of 284 hits obtained from this approach. Among the more abundant encoded proteins identified, several ion channel inhibitors, metalloproteinases, neurotoxins, protease inhibitors, protease activators, cysteine-rich secretory proteins, phospholipases A, and antimicrobial peptides were found. Among the less abundant venom proteins such as growth factors, lipolysis-activating peptides, hyaluronidase, and, phospholipase D also were found (Figure 2).

### 3.3 Identification of LVP1-alpha and LVP1-beta

Using our local database, we conducted exhaustive BLAST searches of the *H. zagrosensis* venom gland transcriptome. We found some transcripts with high sequence similarity to LVP1-alpha and LVP1-beta. Generating a target database of LVP1 sequences from a few widely used databases facilitated the use of comprehensive search strategies to extract subsets of LVP1 sequences from our dataset. In order to increase the sequence identification confidence and to classify the sequences, the obtained sequences were directly searched against NCBI and UniProt databases. Sequences which have a high sequence identity with previously classified LVP1 sequences belonging to the species mentioned above were considered members of this group and subjected to further analysis for classification. We found a total of five LVP1s isoforms in the venom transcriptome of *H. zagrosensis*, three isoforms of the alpha subunit, and two isoforms of beta subunits. All of these five sequences were deposited in GenBank (https://www.ncbi.nlm.nih.gov/) and allocated Accession numbers (Supplementary Table S1). These proteins also are available in the ScorpDb database (https://scorpdb.com/).

For identification of protein domains and/or families, the LVP1 proteins encoded by the venom glands were thoroughly investigated using the HMMER, Pfam, Superfamily, Gene3D, and NCBI Batch CD-Search tools. The InterProScan program was used to validate all data and the annotated functions of the LVP1s based on homologous proteins, indicating the accuracy of performed annotations. The InterProScan results suggest that the VLP1s alpha/beta are classified as non-cytoplasmic domain into Scorpion toxin-like family, and Knottin, scorpion toxin-like homologous superfamily. The result of InterProScan analysis of all data have been presented as a Supplementary Material.

**FIGURE 3:**
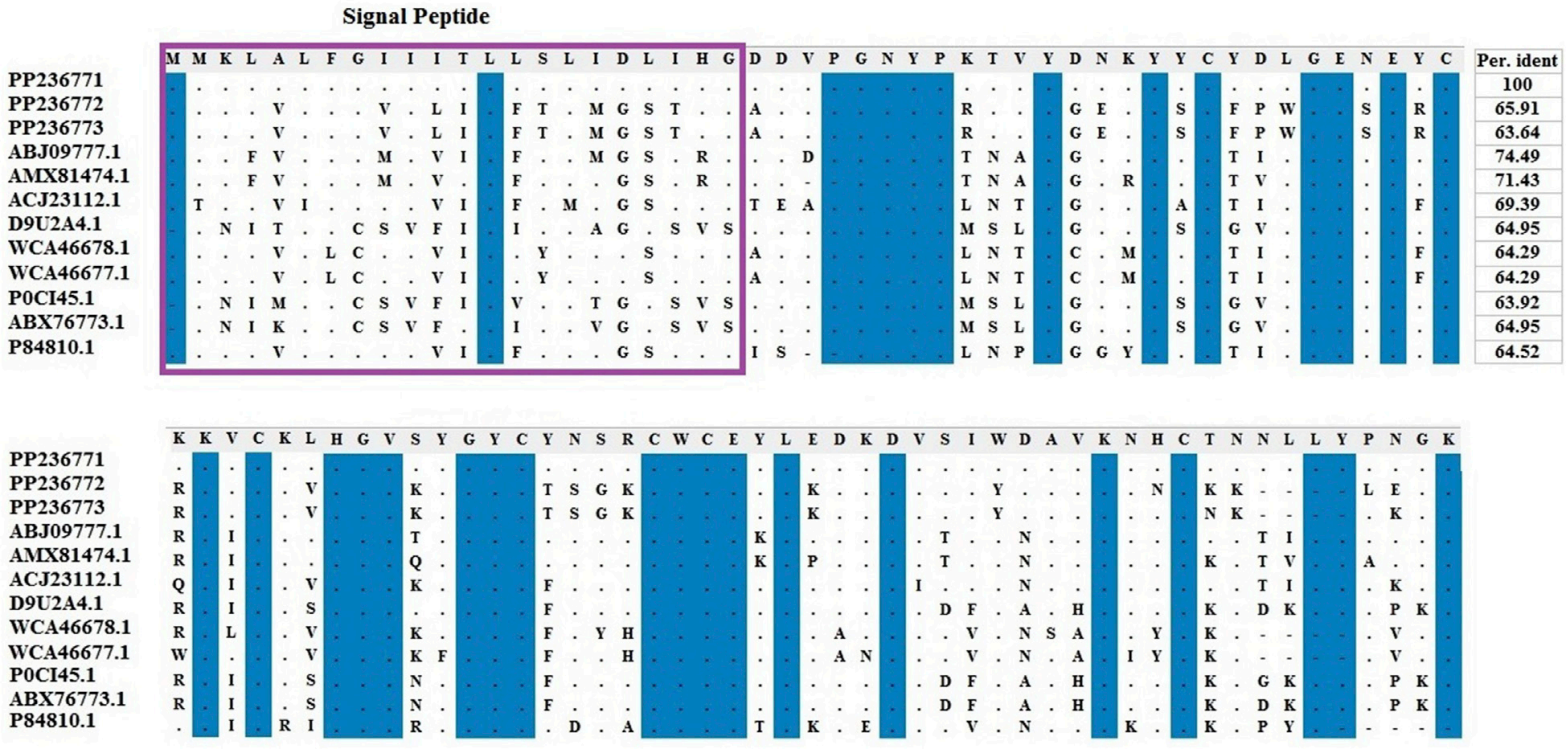
Multiple sequence alignment of the scorpion’s catalytic sequences of LVP1_alpha with MEGA X program. The accession numbers are respectively related to scorpions that are listed as follow: HzLVP1_alpha1, *H. Zagrosensis* (PP236771); HzLVP1_alpha2, *H. Zagrosensis* (PP236772); HzLVP1_alpha3, *H. Zagrosensis* (PP236773); BmLVP1-alpha, *M. martensii* (ABJ09777.1); MeLVP1_alpha1, *M. eupeus* (AMX81474.1); BoiLVP1-alpha*, Buthus occitanus* (ACJ23112.1); AcLVP1-beta2, *A. crassicauda* (WCA46678.1); AcLVP1-beta1, *A. crassicauda* (WCA46677.1); LVP1-alpha*, Lychas mucronatus* (D9U2A4); LV1A2_LYCMC VLP LVP1-alpha, *L. mucronatus* (P0CI45.1); LmNaTx25, *L. mucronatus* (ABX76773.1); BotLVP1-alpha*, B. occitanus tunetanus* (P84810.1). Sequence identities are shown in right. Signal peptides are shown in a purple rectangle.

**FIGURE 4:**
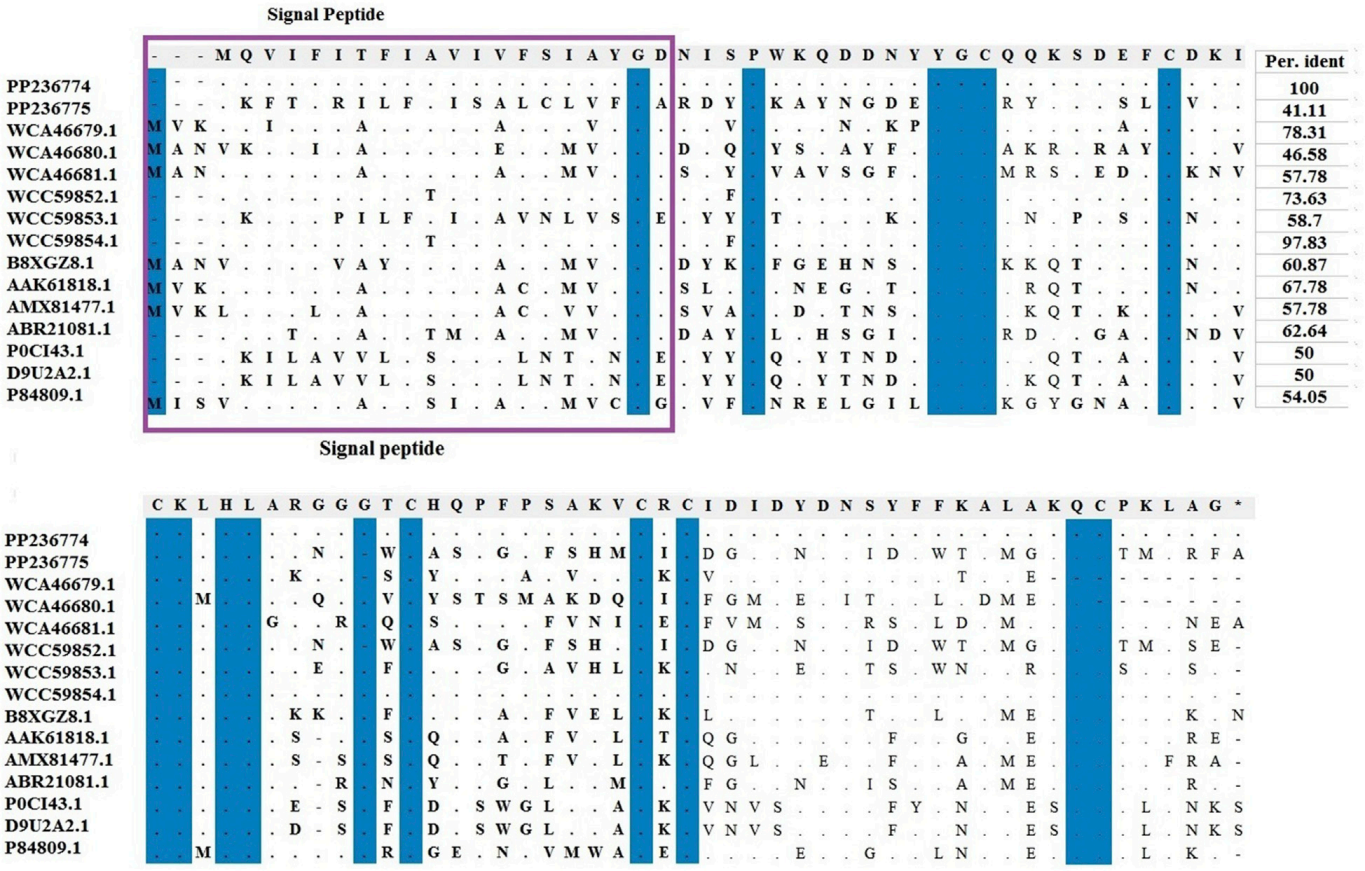
Multiple sequence alignments of scorpion’s catalytic sequences of LVP1_beta with MEGA X program. The accession numbers are respectively related to scorpions that are listed as follow: HzLVP1-beta1, *H. zagrosensis* (PP236774); HzLVP1-beta2, *H. zagrosensis* (PP236775); AcLVP1-beta1, *A. crassicauda* (WCA46679.1); AcLVP1-beta2, *A. crassicauda* (WCA46680.1); AcLVP1-beta3, *A. crassicauda* (WCA46681.1); HsLVP1-beta1, *H. saulcyi* (WCC59852.1); HsLVP1-beta2, *H. saulcyi* (WCC59853.1); HsLVP1-beta3, *H. saulcyi* (WCC59854.1); BoiLVP1-beta, *B. occitanus* (B8XGZ8.1); BmLVP1-beta, *M. martensii* (AAK61818.1); MeLVP1_beta1, *M. eupeus* (AMX81477.1); Venom lipolysis activating peptide beta subunit, *M. eupeus* (ABR21081.1); LVP1-beta, *L. mucronatus* (P0CI43.1); LmNaTx19, *L. mucronatus* (D9U2A2.1); BotLVP1-beta, *B. occitanus tunetanus* (P84809.1). Sequence identities are shown in the right. Signal peptides are shown in a purple rectangle.

Amino acids of identified LVP1-alphas and LVP1-betas from *H. zagrosensis* were aligned with similar peptides from other scorpions and were shown in Figures 3, 4, respectively.

### 3.4 Characterization and 3D-modeling of LVP1s

Physicochemical properties of mature proteins of LVP1 subunits alpha and beta were calculated and listed in Supplementary Table S2. The molecular weight of the identified peptides was between 8146.29 and 10686.56. HzLVP1_alpha2 has the highest molecular weight and HzLVP1_alpha3 has the lowest molecular weight. All of LVP1s have a good water solubility.

The instability index measures the stability of proteins in the experimental conditions. In this study, the instability index of all LVP1s, except for HzLVP1_beta1, were estimated less than 40, indicating that all proteins except for HzLVP1_beta1, are stable (Guruprasad et al., 1990).

The aliphatic index of a protein is a measure of the relative volume occupied by aliphatic side chain of the following amino acids: alanine, valine, leucine and isoleucine. An increase in the aliphatic index increases the thermostability of globular proteins. The aliphatic index values related to LVP1 isoforms of *H. zagrosensis* varies from 42.39 to 71.38.

Negative values of GRAVY (grand average of hydropathicity index) for a protein indicates that it is a hydrophilic protein (Panda and Chandra, 2012). A negative GRAVY was predicted for all identified LVP1s. Accordingly, all LVP1s of *H. zagrosensis* were categorized as hydrophilic proteins.

**FIGURE 5:**
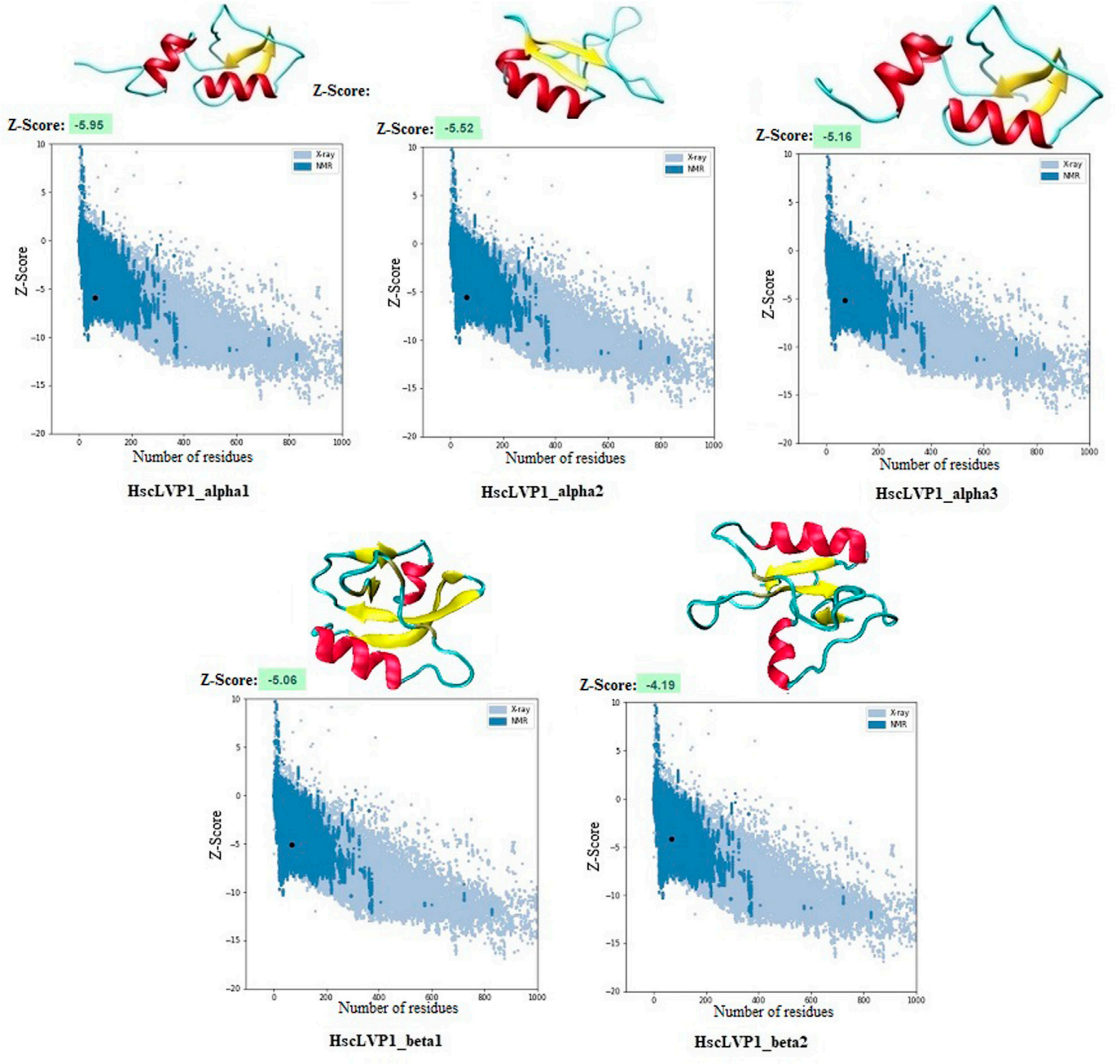
Three-dimensional structure of LVP1s identified in the venom gland of *H. zagrosensis* generated using SWISS-MODEL and I-TASSER. Plots related to validation of each model calculated by Z-score are also shown.

Homology modeling of the identified proteins indicated that the number of alpha-helix and beta-strands as well as the compactness are different in isoforms of LVP1 (Figure 5). The calculated Z-score was used for evaluation the overall quality and measuring the deviation of total energy for the predicted protein structures (Wiederstein and Sippl, 2007). More negative Z-scores indicate more valid structures (Gupta et al., 2013). Measuring the Z-score for determined LVP1 structures yielded all structures with significant Z-scores in the in the range of −4.19 and −5.95 (Figure 5).

**FIGURE 6:**
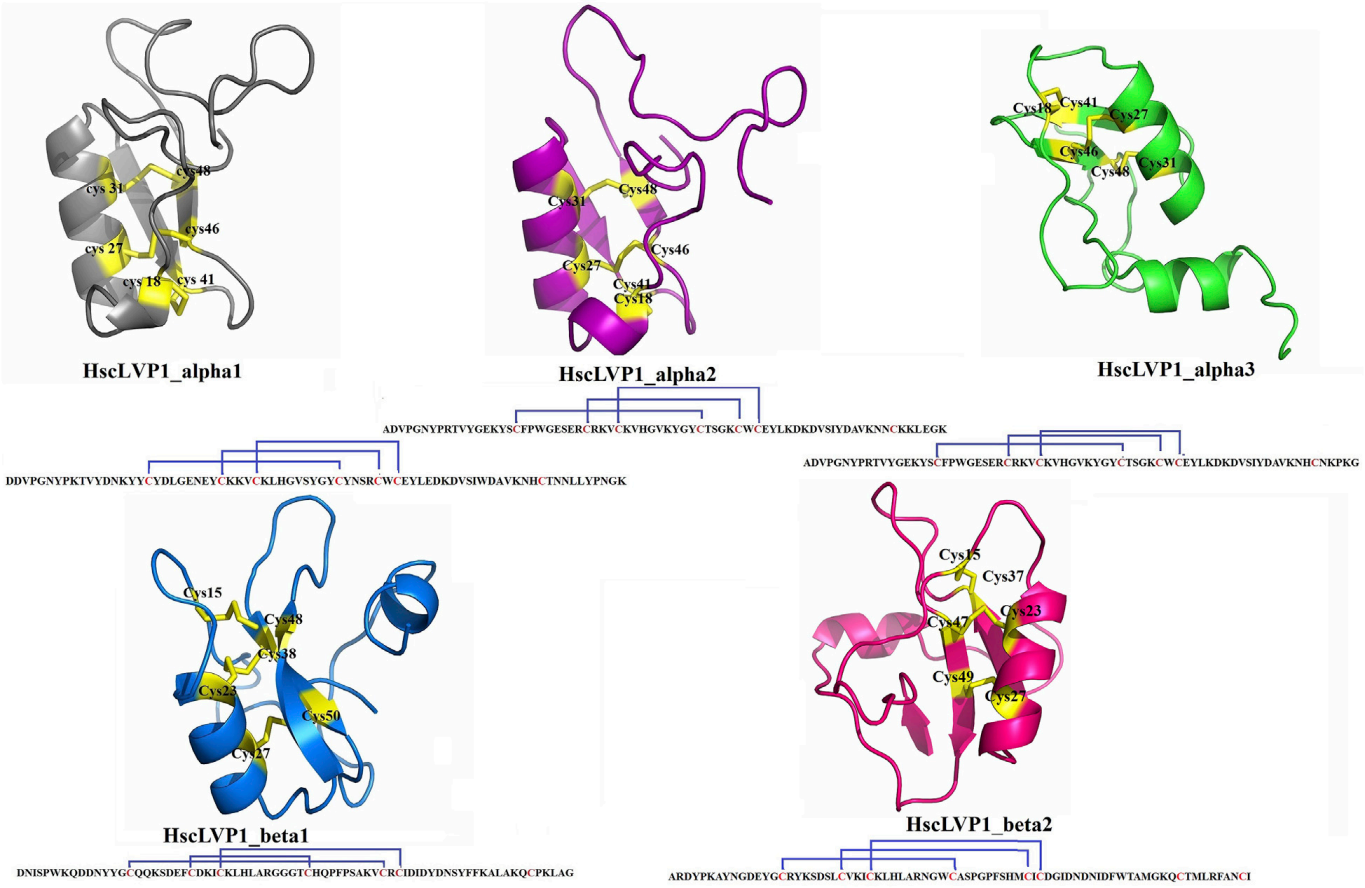
The structure and disulfide bonding pattern of snakin-1 of LVP1s subunits alpha and beta identified in the venom gland of *H. zagrosensis*.

The three-dimensional structures of all identified isoforms of LVP1 are tightly packed by three disulfide connectivity formed between C1-C4, C2-C5, and C3-C6 in HzLVP1_alpha1, HzLVP1_alpha2, and HzLVP1_alpha3; C1-C5, C2-C4, and C3-C6 in HzLVP1_beta1; and C1-C4, C2-C5, and C3-C6 in HzLVP1_beta2 (Figure 6).

### 3.5 Interaction of LVP1s with macromolecules involved in lipolysis regulatory pathway

**FIGURE 7:**
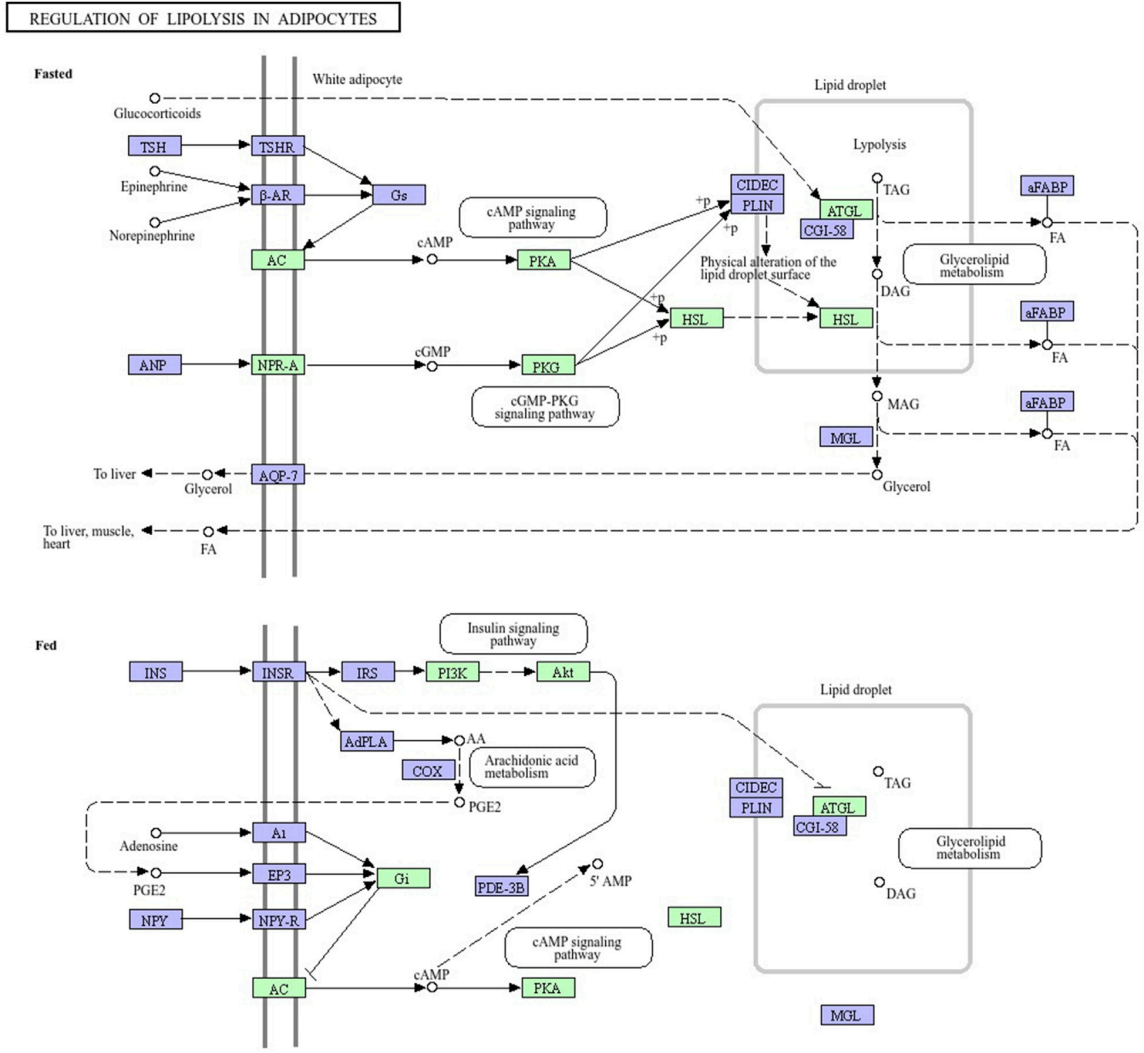
Pathway map of regulation of lipolysis in adipocytes, exported from KEGG. Macromolecules involved in the pathway have shown in green and purple rectangles. LVP1s of *H. zagrosensis* have an interaction with macromolecules presented in green rectangles. ATG, patatin-like phospholipase domain-containing protein 2; HSL, hormone-sensitive lipase; PKA, protein kinase A; PKG1, cGMP-dependent protein kinase 1; ADCY1, adenylate cyclase 1; NPR-A, atrial natriuretic peptide receptor A; AKT, RAC serine/threonine-protein kinase; PIK3K, phosphatidylinositol-4,5-bisphosphate 3-kinase catalytic subunit alpha/beta/delta; Gi, guanine nucleotide-binding protein G (i) subunit alpha.

The mRNAs from *H. zagrosensis* transcriptome that show interaction with LVP1s were searched using RNAPlex v1.6. Interacting mRNAs were subjected to the KEGG to analyze their function. KEGG pathway analysis suggested some pathways that by which LVP1s can interact with macromolecules. A top KEGG pathway enriched by interacted proteins is regulation of lipolysis in adipocytes. Due to the potential for lipolytic activity of LVP1S based on the similarity, the metabolic pathway of regulation of lipolysis in adipocytes was investigated. All molecules involved in this pathway are shown in Figure 7, in which those molecules that interacted with LVP1s of *H. zagrosensis* are shown in a green rectangle. According to this figure, LVP1s can interact with patatin-like phospholipase domain-containing protein 2 (ATG), hormone-sensitive lipase (HSL), protein kinase A (PKA), cGMP-dependent protein kinase 1 (PRKG1), adenylate cyclase 1 (ADCY1), atrial natriuretic peptide receptor A (NPR-A), RAC serine/threonine-protein kinase (AKT), phosphatidylinositol-4,5-bisphosphate 3-kinase catalytic subunit alpha/beta/delta (PIK3K), and guanine nucleotide-binding protein G(i) subunit alpha (Gi).

## 4 Discussion

In addition to being used to produce antivenom, scorpion venoms are studied for a better understanding of the pathological and pathophysiological effects caused by scorpion envenoming and the ability of their low molecular weight proteins to treat diseases (Baradaran, 2023; Krämer et al., 2022). Therefore, it is important to identify the constituents of scorpion venoms. *H. zagrosensis* is one of the endemic scorpion species in Iran. However, the components of its venom have not been studied. In order to further increase the number of identified proteins, we analyzed here the transcriptome of the *H. zagrosensis* venom gland with emphasis on sequence annotations. In the present work, we conducted the first whole-transcriptome analysis of *H. zagrosensis* venom gland that generated ∼ 46 million paired clean reads, resulting in a total of 213,626 contigs with an N50 length of 2,116 bp along with 136,897 unigenes with 20,345 annotated proteins. In the previous research experience we also used a deep sequencing approach and described an initial study of the transcriptomes of four main scorpion species from Iran, including *H. saulcyi*, *A. crassicauda*, *and Hemiscorpius lepturus* (Baradaran and Salabi, 2023), which were assembled using Trinity. The transcriptome analysis of the *A. crassicauda* venom gland was performed with 472 million clean reads pairs and generated 952,725 contigs representing 585,177 unigenes (Salabi et al., 2021b). Similarly, we describe the transcriptome analysis of the venom gland from *H. saulcyi* with 97 million paired clean reads, which generated 191,150 assembled transcripts and 110,126 unigenes (Salabi et al., 2023). In the transcriptome analysis of the venom gland of *Centruroides noxius* more than three million reads were obtained which assembled in 19,000 isogroups. The annotation of this study revealed the presence of important elements of the small non-coding RNA processing machinery and microRNA candidates (Rendón-Anaya et al., 2012).

In the current study, clustering the transcriptome using CD-Hit-EST resulted to generate 101,180 contigs and 96,071 unigenes with an N50 length of 1,149 bp. The BUSCO analysis showed a high BUSCO completeness score of >92%. Here, although high recoveries were obtained for raw and final assemblies, the score of duplicated BUSCOs was significantly reduced after using CD-Hit-EST to generate the final assembly. In study of the transcriptome of *A. crassicauda* assembled by Trinity, also a high BUSCO completeness score (>96%) was reported along with high “duplication” BUSCOs score, in which the clustering step led to decreased the number of duplicated copies of transcriptomes (Salabi and Jafari, 2022). Similarly, in the transcriptome analysis of *Centruroides vittatus* a high BUSCO score of 97.8% was achieved for the final assembly (Yamashita et al., 2024).

In the current study, a total of 5,386 peptides and proteins were predicted. This number of predicted proteins is definitely more than analysis what achieved for *Superstitionia donensis.* The Illumina sequencing analysis of *Superstitionia donensis* was generated a total of 219,073 transcripts, which annotation revealed that 135 transcripts code the peptides similar to known venom components available from different protein databases (Santibáñez-López et al., 2016). The annotation of predicted proteins from *H. zagrosensis* transcriptome in the current study using BLASTp also showed that a large number of predicted proteins had significant similarity with hits available in UniProt, SwissProt, Animal toxin annotation project, and Pfam protein databases. The annotation obtained across those databases and GhostKOALA server also confirmed the accuracy of the assembled transcripts. In the venom gland transcriptome analysis of *Mesobuthus martensii,* also 16,726 (37.47%), 10,076 (22.57%), 10,878 (24.37%), and 10,187 (22.82%) unigenes were found to have similarity with coding proteins from NR, Swissport, GO, and KEGG databases, respectively (Yang Y. et al., 2022).

Several predicted proteins were identified for the first time in this transcriptome, such as several ion channel regulators, metalloproteinases, neurotoxins, proteases, phospholipases, antimicrobial peptides, hyaluronidases, growth factors, and, lipolysis-activating peptides.

One of the important findings of this study was the identification of lipolysis-activating peptides (LVP1s). We found three sequences encoding toxins similar to LVP1 subunit alpha (PP236771, PP236772, and, PP236773) and two scorpion venom toxins similar to LVP1 subunit beta (PP236774 and PP236775), which have been studied in venom gland transcriptomes of *H. saulcyi*, *M. eupeus*, and, *A. crassicauda* (Baradaran and Salabi, 2023). Our previous work on the analysis of the venom gland transcriptomes of *H. saulcyi*, *M. eupeus*, and, *A. crassicauda* (Baradaran and Salabi, 2023), in addition to identifying several important compounds of scorpion venoms, indicated that their venoms contain isoforms of LVP1 subunit alpha and beta.

The Physicochemical analysis of LVP1s subunits alpha and beta originated from *H. zagrosensis* (Supplementary Table S2) indicated that except for HzLVP1_alpha1, all other identified LVP1s of *H. zagrosensis* are basic. Instability index also showed that except for HzLVP1_beta1, all other identified LVP1s are stable proteins (instability index <40). The instability index of HzLVP1_beta1was 48.45. Furthermore, due to negative values estimated for GRAVY, all proteins included in this study were hydrophilic. The additional physicochemical analysis of LVP1s from *H. zagrosensis* showed that their aliphatic index values ranging from 42.39 to 71.38, which indicated that all isoform of LVP1s alpha and beta subunits are thermally stable. However, since that the higher value of aliphatic index indicates more thermostability (Panda and Chandra, 2012), HzLVP1_alpha2 (aliphatic index = 71.38) is the most thermostable LVP1. This value is higher than previous aliphatic index ranging from 30.33 to 54.26 was for neurotoxins (Panda and Chandra, 2012). The HzLVP1_alpha2 has also the highest half-life as well as also the lowest instability index. Therefore, since HzLVP1_alpha2 is more stable than other homolog proteins, we recommend to study more about this protein. Disulfide bridges, commonly found in extracellular proteins, contribute to the stability of the three-dimensional structures. Therefore, they play a critical role in stabilizing folded conformation of proteins (Tsai et al., 2005). According to the presence of cysteine and disulfide bridges, scorpion venom proteins are classified into disulfide bridged proteins (DBPs) and non-disulfide bridge proteins (NDBPs) (Almaaytah and Albalas, 2014). All proteins identified in this study contain 7 cysteine residues, except for HzLVP1_beta2 which has 8 cysteine residues. However, all of them form 3 disulfide bridges. Therefore, there is a free cysteine in the HzLVP1_alpha1, HzLVP1_alpha2, HzLVP1_alpha3, HzLVP1_beta1, and two free cysteines in HzLVP1_beta2. In fact, the two free cysteines in HzLVP1_beta2 are distant, preventing them from interacting. As was discussed before, it seems RNA editing is responsible for changing the position of cysteines in similar proteins (Hoopengardner et al., 2003; Zhu and Gao, 2006). Similar to what we discussed about MeLVP1_apha1, MeLVP1_beta1, AcLVP1_beta3, and HsLVP1_beta1, 2, 3 (Salabi et al., 2023), the six cysteine residues of HzLVP1_alpha1, HzLVP1_alpha2, HzLVP1_alpha3, HzLVP1_beta1 are potentially involved in the formation of three intermolecular disulfide bridges, and the extra cysteine may form an intramolecular disulfide bridge to form a dimer molecule, either a homodimer or a heterodimer. LVP1 in the homodimer form was determined to be an inhibitor of HMG-CoA reductase (Chai et al., 2012), and in heterodimer form induces lipolysis in adipocytes of mouse (Drira-Chaabane et al., 1996; Soudani et al., 2005a). The conformation of two free cysteines in HzLVP1_beta2 are controversial and further research will be necessary to resolve these discrepancies.

For additional analysis, protein–LVP1s interactions involved in regulation of lipolysis in adipocytes was also assessed. These molecules may provide new therapeutic targets (Althaher, 2022; Grabner et al., 2022; Li et al., 2021). Figure 7 shows that LVP1 subunits of *H. zagrosensis* potentially interact with some macromolecules, including adipose triglyceride lipase (ATGL) and hormone-sensitive lipase (HSL), which have key roles in the regulation of lipolysis (Schweiger et al., 2006). Previously it has been reported that lipolysis in adipocytes depends on these two lipases, ATGL and HSL (Figure 7) (Schweiger et al., 2006). ATGL initiates degradation of triglycerides (TAG), and then with the intervention of HSL, it converts to monoacylglycerol and fatty acids (FAs), and intramyocardial triglyceride levels reduce in the heart and improve myocardial function. Dysfunction of lipolytic enzymes results in a change in release of free fatty acids (FFAs), leading to clinical diseases including obesity, liver steatosis, cancer, and cardiomyopathy6 (Schweiger et al., 2006). Thus, inactivated ATGL is highly relevant to diseases in mice and humans (Rajani et al., 2020; Raje et al., 2020; Yang and Mottillo, 2020). On the other hand, increasing the activity of ATGL leads to the increase of plasma FFA which can promote ectopic lipid deposition, IR, as well as vascular and cardiac dysfunction (Henderson, 2021). So, ATGL is known as an interesting pharmacological target (Schreiber et al., 2015; Schweiger et al., 2017). Pharmacological activators and inhibitors of ATGL have considered by researchers (Li et al., 2021). For example, Atglistatin is a mouse-selective ATGL inhibitor which protects from high-fat diet-induced insulin resistance, liver inflammation, and liver steatosis. Another is a small human selective inhibitor, NG-497, which decreases FFA in human adipocytes in a reversible manner (Grabner et al., 2022). Nature can also be a resource for ATGL inhibitors. Oroxylin A is a natural flavonoid extraction from the root of *Scutellaria baicalensis* Georgi which prevents ameliorating hepatic fibrosis (Chen et al., 2018; Jin et al., 2018), hepatic steatosis (Jin et al., 2018), and promoting liver regeneration (Zhu et al., 2013). The activity of ATGL is stimulate with CGI-58 by up to 20-fold (Lass et al., 2006). Metabolic pathway analysis (Figure 7) demonstrated that LVP1s of *H. zagrosensis* potentially interact with ATGL suggesting that they can inhibit or activate this enzyme which in both ways can be a candidate as promising drugs.

HSL also may provide a new pharmacological target to reduce FFA levels in plasma. HSL inhibitors are currently developing for drug design and usage to treat dyslipidemia and insulin resistance and blood glucose handling in type II diabetes (Althaher, 2022). Various HSL inhibitors have been identified from natural resources like plants and microbes (Bustanji et al., 2010; Jawed et al., 2019; Vértesy et al., 2002; Yang X. D. et al., 2022). According to the pathway analysis performed in the current study (Figure 7), LVP1s of *H. zagrosensis* interact with HSL, suggesting that LVP1s of *H. zagrosensis* may provide a new opportunity as HSL inhibitors.

## 5 Conclusion

Transcriptome analysis of the venom gland of *H. Zagrosensis* revealed the presence the five isoforms of alpha and beta subunits of LVP1 protein in the venom gland of this scorpion. Physicochemical analysis described here determined that all identified isoforms of LVP1 are hydrophilic, and although apart from HzLVP1_beta1, all of them except are stable, HzLVP1_alpha2 is the most stable isoform. The three-dimensional structure of all identified isoforms of LVP1 is characterized by three distinct disulfide bonds stabilizing the protein’s tertiary structure. Moreover, functional analysis of LVP1s indicated the interaction of LVP1s with major enzymes involved the catabolism of adipocyte tissue triacylglycerol, suggesting a pharmacological property for these proteins. However, further research may help to better understand about the exact functions of these proteins.

## Data Availability

The datasets presented in this study can be found in online repositories. The names of the repository/repositories and accession number(s) can be found below: https://www.ncbi.nlm.nih.gov/genbank/, PP236771, https://www.ncbi.nlm.nih.gov/genbank/, PP236772, https://www.ncbi.nlm.nih.gov/genbank/, PP236773, https://www.ncbi.nlm.nih.gov/genbank/, PP236774, https://www.ncbi.nlm.nih.gov/genbank/, PP236775.
