# Supplementary material for: First transcriptome analysis of the venom glands of the scorpion Hottentotta zagrosensis (Scorpions: Buthidae) with focus on venom lipolysis activating peptides
Source: Front Pharmacol. 2024 Nov 13;15:1464648. doi: 10.3389/fphar.2024.1464648 (PMC11598519; doi:10.3389/fphar.2024.1464648)
Supplement: Supplementary file 1 [file DataSheet1.docx]

**
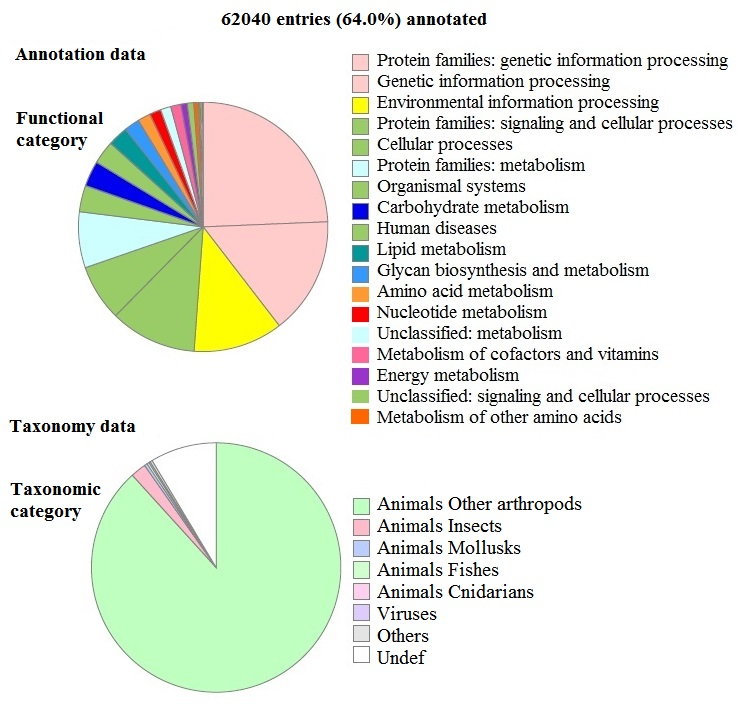
**

**Figure S1.** Output summary of functional distribution of protein-coding genes and taxonomic information in the GhostKOALA server.

**Table S1.** Identified and submitted isoforms of LVP1-alpha and LVP1-beta in GeneBank database.

| Protein name | Accession number |
| --- | --- |
| HzLVP1_alpha1 | PP236771 |
| HzLVP1_alpha2 | PP236772 |
| HzLVP1_alpha3 | PP236773 |
| HzLVP1_beta1 | PP236774 |
| HzLVP1_beta2 | PP236775 |

HzLVP1_alpha1: *H. zagrosensis* Lipolysis activating peptide 1_alpha subunit isoform X1

HzLVP1_alpha2: *H. zagrosensis* Lipolysis activating peptide 1_alpha subunit isoform X2

HzLVP1_alpha3: *H. zagrosensis* Lipolysis activating peptide 1_alpha subunit isoform X3

HzLVP1_beta1: *H. zagrosensis* Lipolysis activating peptide 1_beta subunit isoform X1

HzLVP1_beta2: *H. zagrosensis* Lipolysis activating peptide 1_beta subunit isoform X2

**Table S2.** Physicochemical properties of LVP1_alpha and LVP1_beta of *H. zagrosensis*.

| Peptide name | Signal peptide (bp) | Mature peptide (bp) | Molecular weight (g/mol) | Theoretical pI | Net charge at pH 7 | Water solubility | Instability index | Aliphatic index | Grand average of hydropathicity (GRAVY) | Estimated half-life |
| --- | --- | --- | --- | --- | --- | --- | --- | --- | --- | --- |
| HzLVP1_alpha1 | 22 | 76 | 8983.01 | 5.53 | -2.3 | Good | 23.59 | 55.00 | -0.886 | -1.1 hours (mammalian reticulocytes, in vitro).  -3 min (yeast, in vivo).  >10 hours (Escherichia coli, in vivo) |
| HzLVP1_alpha2 | 22 | 94 | 10686.56 | 8.97 | 5.7 | Good | 14.78 | 71.38 | -0.235 | -30 hours (mammalian reticulocytes, in vitro).  ->20 hours (yeast, in vivo).  ->10 hours (Escherichia coli, in vivo). |
| HzLVP1_alpha3 | 22 | 71 | 8146.29 | 8.85 | 4.7 | Good | 19.04 | 42.39 | -0.852 | -4.4 hours (mammalian reticulocytes, in vitro).  ->20 hours (yeast, in vivo).  ->10 hours (Escherichia coli, in vivo). |
| HzLVP1_beta1 | 19 | 73 | 8295.40 | 7.72 | 0.7 | Good | 48.45 | 53.36 | -0.677 | -1.1 hours (mammalian reticulocytes, in vitro).  -3 min (yeast, in vivo).  ->10 hours (Escherichia coli, in vivo). |
| HzLVP1_beta2 | 19 | 76 | 8604.88 | 7.73 | 0.7 | Good | 27.60 | 57.89 | -0.328 | -4.4 hours (mammalian reticulocytes, in vitro).  ->20 hours (yeast, in vivo).  ->10 hours (Escherichia coli, in vivo). |
